# The Effectiveness of Adapted Personalized Motor Activity (AMPA) to Improve Health in Individuals with Mental Disorders and Physical Comorbidities: A Randomized Controlled Trial

**DOI:** 10.3390/sports10030030

**Published:** 2022-02-25

**Authors:** Vito Lamberti, Stefano Palermi, Andrea Franceschin, Giovanni Scapol, Vincenzo Lamberti, Chiara Lamberti, Marco Vecchiato, Rocco Spera, Felice Sirico, Elisabetta Della Valle

**Affiliations:** 1 Sport Medicine and Motor Activity Institute c.FMSI-CONI, Vittorio Veneto, 31029 Treviso, Italy; vitorbras7@hotmail.it (V.L.); andreafranceschin@libero.it (A.F.); gioscapol@gmail.com (G.S.); direzione@centromedicinadellosport.it (V.L.); chiarapuntolamberti@gmail.com (C.L.); 2 Department of Public Health, University of Naples Federico II, Via S. Pansini 5, 80131 Naples, Italy; rocco.spera@unina.it (R.S.); sirico.felice@gmail.com (F.S.); elisabetta.dellavalle@unina.it (E.D.V.); 3 Sports and Exercise Medicine Division, Department of Medicine, University of Padova, 35100 Padova, Italy; marcovecchiato.md@gmail.com

**Keywords:** exercise-therapy, exercise physiology, physical activity, mental health, physical comorbidities

## Abstract

Mental disorders are highly prevalent worldwide and have a high impact on daily functioning. Exercise therapy was found to improve health of individuals with physical and mental disorders. This study aims to investigate the effectiveness of an Adapted Personalized Motor Activity (AMPA) in improving health in individuals with physical and mental disorders. Forty-three patients affected by both mental and chronic nontransmissible conditions were randomly assigned to intervention group (AMPA intervention) and control group (no intervention). Perceived physical and mental health were assessed using the Short Form 12 (SF-12) questionaries. Moreover, subjects underwent an accurate medical screening process, complete clinical evaluation, body composition evaluation, and cardiopulmonary assessment. Repeated Measurement Analysis of the Variance (RM-ANOVA) was used to compare any changes in health and physiological parameters in-between groups. AMPA group showed a statistically significant improvement in both perceived mental and physical health. Moreover, Body Mass Index (BMI), glycolipid profile, aerobic functional capacity and cardiopulmonary parameters improved significantly among individuals from the intervention group compared with the individuals from the control group. AMPA may be considered a possible intervention to improve health in individuals suffering from multiple physical and mental disorders. Future studies should examine the effectiveness in larger and heterogeneous sample of chronically ill patients and the long-term effect of AMPA.

## 1. Introduction

Mental disorders are among the most frequent diagnoses worldwide [1] and have a tremendous impact on quality of life [2], being a leading cause of disability [3]. According to the global burden of diseases [4], the number of years lost due to disability related to mental conditions increased by almost 50% in the last three decades.

A consistent and reciprocal association between physical and mental health was proved [5]. Moreover, mental disorders are associated with worst health outcome among individuals affected by a wide range of physical diseases [6].

During the last years, growing attention was given to physical exercise as an effective tool in the prevention and treatment of noncommunicable diseases and in the improvement in quality of life [7,8]. Exercise therapy was shown to have benefits in a wide range of chronic physical conditions [9,10,11]. More recently, physical activity was found to be beneficial also in the treatment of individuals with mental disorders [12]. For example, regular physical activity programs were associated with a decrease in depressive symptoms in individuals with mental disorders and in the symptoms of schizophrenia [12]. Exercise therapy was found to be comparable to pharmacological and psychotherapy care in the treatment of moderate anxiety and/or depressive disorders [13], while for severe mental disorders, it still represents a valuable complementary therapy [13]. Furthermore, physical activity slows the physiological cognitive decline associated with age and has a favorable impact on cognitive decline in patients with dementia diagnosis (e.g., Alzheimer’s disease [14]). Some authors suggested that the use of motor rehabilitation since the early stages of the psychopathological path of dementia might be beneficial [15].

Therefore, exercise therapy may represent an effective integrated approach for the treatment of patients suffering from mental disorders and physical comorbidities: it could be helpful to tackle the vicious circle of mental illness and physical diseases, given the fact that one might cause the other and vice versa.

The use of exercise therapy in the treatment of individuals suffering from mental disorders gained considerable interest both in the psychotherapy and in sport medicine areas [16]. However, the variability of the physical activity interventions proposed by different studies make the generalization of results difficult. Even if physical activity determines significant improvements in different body systems, it has inherent risks that can lead to paradox negative effects. Therefore, it is necessary to customize the intervention based on the characteristics of the patients. To this purpose, in the field of sport medicine, promising results were obtained with Adapted Personalized Motor Activity (AMPA), adequately prescribed according to the functional needs of the patients [16]; in particular, it determines a targeted approach to the problems of the subject. While the effectiveness of AMPA in improving conditions of patients with chronic degenerative diseases were previously proven [16], no information is available concerning the benefits of this exercise therapy in patients with both mental disorders and physical comorbidities.

Therefore, the aim of this study was to investigate the impact of an AMPA program on perceived mental and physical health in patients suffering from mental disorders and chronic nontransmissible conditions. Anthropometric, cardiopulmonary and blood parameters were investigated too.

## 2. Materials and Methods

### 2.1. Subjects

A convenience sample of 43 subjects was selected among patients from the territorial mental health department. The inclusion criteria were: (i) aged between 40 and 75; (ii) had at least one moderate or severe mental health disorder (major depressive disorder, anxious-depressive disorder); and (iii) had one or more chronic nontransmissible conditions (metabolic syndrome, cardiovascular disease, respiratory disease).

The exclusion criteria were history of musculoskeletal, neurological, or orthopedic disorders within the preceding six months that possibly affected their ability to execute the experimental protocol.

Individuals were randomly (one-to-one) assigned to experimental group (AMPA intervention) or control group (no intervention). Random allocation software (STATA, v.8, College Station, TX, USA) was used to allocate the 43 participants to each of the 2 study groups, using blocks of 4.

The study was carried on in accordance with the principles of the Declaration of Helsinki and it was supported by the Department of Prevention, Service for Education and Promotion of Health of ULSS 7 of Pieve di Soligo (Treviso, Italy). In accordance with the applicable legal regulations and the Code of Medical Ethics, legal/tutor subjects were duly informed on the risks, benefits, and stress deriving from the physical exercise and signed an informed consent form.

### 2.2. Experimental Design

In the first part of the study, subjects underwent a medical screening process, made of anamnesis collection, complete clinical evaluation, body composition evaluation, blood test parameters, and cardiopulmonary assessment. 

Age and sex of each subject were collected. Patients were told to avoid heavy foods and physical activity in the day before the measurement sessions. All the measurements were performed in the morning in a room with a standardized temperature by the same trained examiner. Body weight (kg) was measured in underwear to the nearest 0.1 kg (Tanita Corporation, Tokyo, Japan). Height (cm) was measured with a stadiometer to the nearest 0.5 cm. Both measurements were used to calculate Body Mass Index (BMI, kg/m^2^). Blood tests values investigated were glycated hemoglobin (HbA1c, mg/dL), random glycemia (glycemia, mg/dL), total cholesterol (col TOT, mg/dL), HDL cholesterol (HDL, mg/dL), LDL cholesterol (LDL, mg/dL), and triglycerides (Tg, mg/dL).

The cardiopulmonary status was investigated at rest and during a cardiopulmonary cycle ergometer stress test (V-Max, Ergo 800 S and 12-lead ECG recorder Max-1; Sensor Medics, Yorba Linda, CA, USA). Maximum oxygen uptake (VO_2_ peak, mL/kg/min) was assessed. The system was calibrated before and verified following each test. A 12-lead electrocardiogram was monitored throughout the test, and heart rate was measured beat-by-beat from the R-R interval. Arterial O_2_ saturation was measured from a finger by pulse oximetry (Biox 3745, Ohmeda, Louisville, KY, USA), and blood pressure using a sphygmomanometer. Ratings of perceived exertion were measured during exercise using 1–10 Borg scale. Each test was performed applying a 25 W × 1 min ramp protocol [17] to achieve peak of exercise in 10 ± 2 min, while a respiratory exchange ratio of 1.1 was used to consider the test as maximal. All testing occurred between 7:00 AM and 12:00 AM in a temperature-controlled laboratory. Spirometry (Mir Smart One, Rome, Italy) was used to obtain forced vital capacity (FVC, L), forced expiratory volume in one second (FEV1, L), Tiffenau index (IT: FEV1/FVC, %).

In case of need, further specialist consultants were requested.

### 2.3. Physical and Mental Health Assessment

Perceived physical and mental health were assessed using the Short Form 12 (SF-12) of the 36 items Health Survey [18]. The scale investigates health status and includes two different domains: the Physical Component Summary (PCS) and the Mental Component Summary (MCS). The PCS was used as measure of perceived physical health, while the MCS was used as indicator of perceived mental health. The score of each domain ranges from 0 (worst) to 100 (best).

### 2.4. Training Protocol

After the physiological measurements, the sports medicine physician prescribed the “dose range” of exercise for each participant, according to their medical condition, and following American College of Sport Medicine (ACSM) recommendations [19]. Based on medical prescription, the sports and exercise science graduate elaborated a “tailored” training plan for each patient. Gym machines (Air Machine–Panatta Sport, Macerata, Italy) were set with each patient’s training plan and used to monitor the sensitive data of the workout, since these machines were connected to each other through an automatic computerized system (Net Tutor Pro software, Rome, Italy). Moreover, the physician could monitor instantly the physiological parameters of the patient and stop the exercise if something went wrong.

### 2.5. AMPA System

Individuals included in AMPA intervention group were subjected to a specific training program.

First, they attended an aerobic reconditioning training period, made by a one-month, low-intensity, and twice-a-week workout (25–35% of the VO_2_ peak and 30–50% of the maximum HR limited by symptoms). 

Subsequently, a nine-month-long personalized training plan was prescribed, with thresholds based on ACSM recommendations [19]: it was composed by both aerobic and anaerobic (strength) workouts. Aerobic training was a medium-intensity training (between 55–75% of the VO_2_ peak and 60–80% of the maximum HR limited by symptoms), made by constant cardiovascular commitment and/or interval training. It was carried out with the use of cardio machines (Treadmill, Elliptical, Upright bike, Recumbent bike). Strength training was conducted by a variable load of 50–70% of the maximum voluntary muscle contraction (with a relative increase in the heart rate, which was always lower than 70% of the maximum heart rate limited by the symptoms). It was carried out with the use of compressed-air isotonic machines (ercolina, pectoral machine, lower-back, leg-press, adductor machine, deltoids press, lat pulldown convergent). These guarantee the correct progression of work and observance of the necessary time intervals thanks to the ability to vary the lower load to 2 kg [20]. For exercise set (on average 3 sets), 8–15 repetitions were performed, with a rest interval between sets of 1–3 min [21,22].

All sessions were carried out at the same venue and at the same time of the day (11 a.m.) to avoid any circadian effects. Patients underwent 3 training sessions per week, composed of 25–30 min of aerobic exercises and 20–30 min of strength exercises. Therefore, each of the three weekly workouts had a total duration of 50–60 min. Each training was always preceded by 5–10 min of free body activity and terminated with a suitable recovery phase.

Subjects allocated in the control group were not included in structured programs of physical activity.

### 2.6. Statistical Analysis

Quantitative variables were summarized as mean (m) and standard deviation (SD). Normal distribution was assessed through Shapiro–Wilk test. Frequencies of comorbidities were compared between groups through chi square test. For frequencies less than 5, Fisher correction was applied. If a frequency of 0 was recorded in one group (case or control), chi square test was not applicable (NA).

The main outcome measure were the physical component summary (PCS) and the mental component summary (MCS) domains of the SF-12. Differences in anthropometric data, blood test results, exercise stress test, and spirometry were compared too between groups and secondary outcome. Data were analyzed according to a two-way repeated measure ANOVA, with 1 between-subjects factor (treatment group) and 1 within-subjects factor (time of assessment, namely, pre- and postintervention assessment). Interaction between treatment group and time of assessment was investigated as well. Posthoc pairwise comparisons with Bonferroni’s adjustments were not applicable because only two timepoints were recorded (pre- and post-treatment).

A *p*-value of <0.05 was considered statistically significant. The analysis was carried out with STATA software (STATA, v.8, Rome, Italy).

## 3. Results

Data of 43 subjects were collected for the present study. The intervention group was composed of 21 subjects (11 M, 10 F; 57 ± 9 aged), while control group was composed of 22 subjects (10 M, 12 F; 61 ± 8 aged).

All patients in both groups had a confirmed diagnosis of at least one mental disorder and at least one chronic nontransmissible conditions. No significative differences were recorded between groups about the frequencies of specific comorbidities (Table 1).

**Figure 1:**
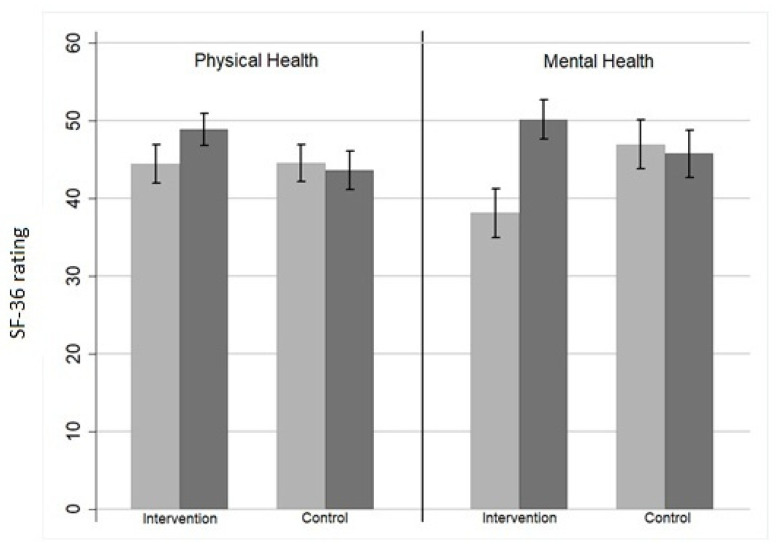
Physical and mental health among individuals from AMPA—intervention (n = 21) and control group (n = 22)—before (light grey) and after (dark grey) intervention. Y-axis indicates rate of Short Form 12 (SF-12) of 36-item Health Survey. (A higher value in “physical health” and “mental health” means that perceived status of physical and mental health in experimental group improved after training protocol).

Both perceived mental and physical health improved significantly in AMPA group compared to that of the control group. Mental health score changed from 38.1 ± 7.4 in the preintervention evaluation to 50.1 ± 5.9 in the postintervention evaluation, while physical health score changed from 44.4 ± 5.8 to 48.8 ± 4.8 (Table 2, Figure 1).

After the intervention, BMI decreased significantly in AMPA group compared to that of the control group. Individuals of AMPA group had a significant reduction in total cholesterol, triglycerides, and LDL values, while there was an improvement in HDL levels, after completing the AMPA protocol. Details are showed in Table 3. Intergroup differences of Hb1Ac are less marked, with a trend that approaches predefined significance levels (*p* = 0.055). Significant difference in cardiopulmonary parameters between the two groups were found: a significant increase in VO_2_ peak was observed. Moreover, a significant improvement in spirometer’s parameters was also obtained.

## 4. Discussion

Results of present study show a significant improvement in self-reported mental and physical health in patients with mental disorders and chronic nontransmissible conditions treated with AMPA program, compared to those from the control group. Patients in AMPA group also showed a significant improvement in anthropometric, cardiopulmonary and blood test parameters.

We found highly significant intergroup improvement both in mental and physical health among individuals after AMPA therapy compared with that of the control group. Numerous studies [23,24] demonstrated that physical activity could improve mental and physical health and quality of life in patients with chronic mental illness undergoing motor re-education programs. Most of the available evidence about the effect of structured and supervised physical activity are results of observational studies or small experimental trials with individuals affected only by one mental pathology [25,26]. In daily practice, sports physicians must work with patients with multiple pathologies who are in need of a comprehensive exercise prescription. The AMPA project was developed as a personalized approach for patients suffering from multiple conditions [16]. Our results confirm the benefits induced by AMPA in patients suffering from at least one mental and one physical condition. The improvements in mental and physical health may be related to a complex series of psychological, cardiovascular, respiratory, metabolic, and muscular adaptations [27]. Specific exercise protocols can have beneficial effects on quality of life, the perception of the state of health, the sense of control of the disease, the reduction in exacerbations and hospitalizations, the diminution in medical consults [28]. Furthermore, for people with mental disorders, the marked improvement in psychological well-being helps with feelings of frustration and isolation, with evident improvement in anxious and depressive states [29,30].

Regarding anthropometrics, we observed a significant decrease in BMI compared to that of the control group. Our findings are in line with previous studies, highlighting a positive dose–response correlation between weight loss- or exercise-induced BMI decrease in overweight people [31], without calorie restriction [32]. The beneficial effects of physical exercise seem even stronger in preventing further increases in bodyweight, maintaining weight loss after calories restriction, and countering the loss of muscle mass during a diet.

The AMPA protocol was also followed by an improvement in the glycolipid profile in terms of total cholesterol, LDL, HDL, and triglycerides, compared to a substantial stationarity or a slight improvement in these values in the control group. Our findings may have important consequences in terms of risk of mortality due to cardiovascular diseases [33], regardless of weight loss. Our data confirm exercise therapy may represent a strategy to improve the lipid profile, together or as an alternative to the prescription of lipid-lowering drugs [34]. The underlying mechanism appears to be linked to the increase in the oxidation capacity of lipids by muscles, thanks to the fatty acid-mediated activation of enzymes and transport proteins in the skeletal muscle tissue [9].

The improved metabolic capacity of muscle tissue also induces the enhancement in insulin sensitivity, thus explaining the significant decrease in blood glucose detected in this group of patients. The validity of structured physical activity on the glycemia control in diabetic patients is also well documented in literature and constitutes one of the main therapeutic modalities of type 2 diabetes [9,35]. In line with these findings, we observed a significant decrease in the average pre- and postintervention blood glucose values in the experimental group. This demonstrates the clinical relevance of exercise in short-term blood sugar control, with consequent reduction in diabetic complications. Unexpectedly, compared to that of the aforementioned literature, intergroup differences in the average pre- and postintervention Hb1Ac values appeared weaker. Resistance exercise has a much greater effect than aerobic activity on short- and medium-term glycemic control [36]. AMPA program proposed a mixed exercise program, with aerobic training prevalence as a consequence of a prescriptive adapted choice and of the presence of muscle-joint disorders that greatly reduce the use of electromedical strength machines in these subjects. The exercise-induced improvement in insulin sensitivity and lipid profile justifies the reduction in the risk of death from cardiovascular disease [37].

We observed a significant intergroup improvement in the aerobic functional capacity [38]. Furthermore, from the analysis of examined variables, we observed an improvement in the cardiovascular function, both at rest and during cardiopulmonary stress test, which may result in a decreased risk of death from cardiac events [39,40].

The AMPA protocol was associated to a significant improvement in respiratory function. This is extremely important for the management of patients suffering from mental disorders associated with respiratory conditions, such as chronic obstructive pulmonary disease (COPD) and asthma. Furthermore, in the literature, motor re-education is considered an important component in the management of these patients, since with the worsening of the disease, even drug therapy does not always appear to be effective [41]. Exercise therapy, thanks to its ability to relieve dyspnea and a sense of fatigue in daily activities, is effective in improving the psychophysical well-being and quality of life of these subjects [42], as well as through the use of a respiratory rehabilitation group of exercises [43].

The use of AMPA protocol could modify the therapeutic management of mental diseases, often focused on drugs only, leading to a decreased BMI and an improvement in the glycolipid and cardiorespiratory profile, while also improving mental and physical health.

In this regard, the integration and close collaboration between the medical gym work, the motivational support strategy, and the muscle–joint rehabilitation is also important. This aim to prevent exercise-related physical health problems (muscle–joint pain, joint mobility deficiency: i.e., muscular injuries [44] or peripheral nerve lesion [45]) and some common aspects of mental disorders (loss of interest, generalized asthenia, low self-esteem, and self-confidence, fear of moving, etc.) that can interfere with adherence to exercise programs.

Admittedly, this study suffers from a major limitation: the sample was limited to only 43 individuals. Despite the significant results, studies with larger samples are needed to confirm the effectiveness of the AMPA therapy in the treatment of patients with mental and physical comorbidities. Moreover, since no follow up was performed, it was not possible to track the stability of the improvement in BMI, glycolipid and cardiorespiratory profiles, or physical and mental health over time. Longitudinal studies are needed to prove the long-term benefits of AMPA therapy. On the other side, the use of a broadly used and internationally validated questionnaire to assess self-rated mental and physical health represents a strength of our study.

## 5. Conclusions

AMPA protocol should be considered as an effective therapeutic tool for the treatment of patients affected by both mental and physical conditions.

Future studies should examine an even larger and heterogeneous sample of chronically ill patients with a multidisciplinary approach, increasing the length of the intervention and introducing patient follow-up for a better assessment of the outcomes. A key research direction could be to investigate possible correlations between mental and physical health improvements.

## Figures and Tables

**Table 1:** Frequencies of comorbidities in both groups.

Comorbidities		AMPA Group (n = 21)	Control Group (n = 22)	*p* Value
	Metabolic disorders			
Metabolic syndrome (all criteria fulfilled)		12	14	0.66
Hypercholesterolemia (>200 mg/dL)		6	3	0.22
Hypertriglyceridemia (>150 mg/dL)		1	0	NA
Overweight/Obese (BMI > 25 kg/m^2^)		6	2	0.10
Type II DM		0	1	NA
	Cardiovascular diseases			
High Blood Pressure (>130/85 mmHg)		2	6	0.13
Coronary artery disease		3	1	0.27
Arrhythmia		3	0	NA
Valvular disease		0	1	NA
Peripheral artery disease		1	0	NA
Musculoskeletal problems (painful syndrome)		17	16	0.52
COPD		2	1	0.52
Urinary disease		2	1	0.52
Thyroid disease		1	2	0.58

BMI = Body Mass Index; DM = diabetes mellitus; COPD = chronic obstructive pulmonary disease; NA = chi square test not applicable.

**Table 2:** SF12 improvement between groups.

	AMPA Group		Within-Subjects Factor (Time) AMPA Group (Before-After)	Control Group		Within-Subjects Factor (Time) Control Group (Before-After)	Between-Subjects Factor (Group)	Interaction Factor (Group × Time)
Parameter (Unit)	BeforeM (SD)	After M (SD)		Before M (SD)	After M (SD)			
MCS-SF12	38.1 (7.4)	50.1 (5.9)	0.002	46.9 (7.5)	45.7 (7.3)	0.156	0.004	0.001
PCS-SF12	44.4 (5.8)	48.8 (4.8)	0.003	44.5 (5.7)	43.6 (6.0)	0.113	0.002	0.002

MCS = Mental Component Summary; PCS = Physical Component Summary.

**Table 3:** Secondary outcomes: anthropometric, blood tests, cardiopulmonary, and spirometry changes between groups.

	AMPA Group		Within-Subjects Factor (Time) AMPA Group (Before-After)	Control Group		Within-Subjects Factor (Time) Control Group (Before-After)	Between-Subjects Factor (Group)	Interaction Factor (Group × Time)
Parameter (Unit)	Before M (SD)	After M (SD)		Before M (SD)	After M (SD)			
BMI (kg/m^2^)	30.0 (5.8)	29.0 (4.9)	0.001	27.2 (4.9)	27.6 (5.1)	0.156	0.002	0.002
Hb A1C (mmol/mol)	38.9 (5.2)	38.0 (4.9)	0.055	40.6 (11.2)	43.9 (16.8)	0.163	0.023	0.051
Glycemia (mg/dL)	110.2 (10.4)	103.8 (9.2)	0.004	112.00 (23.8)	122.9 (39.3)	0.211	0.004	0.003
Col TOT (mg/dL)	225.7 (33.8)	195.8 (27.4)	0.001	200.6 (32.5)	212.2 (44.3)	0.314	0.001	0.002
HDL (mg/dL)	49.2 (11.1)	55.2 (11.9)	0.004	50.9 (4.9)	49.4 (10.6)	0.451	0.002	0.003
LDL (mg/dL)	141.7 (35.8)	115.4 (23.8)	0.003	122.1 (26.6)	132.7 (38.6)	0.123	0.003	0.004
Tg (mg/dL)	181.0 (64.3)	140.2 (43.3)	0.001	148.0 (57.8)	148.0 (57.8)	0.311	0.002	0.001
VO_2_ peak (mL/kg/min)	19.1 (4.6)	23.0 (4.4)	0.001	18.9 (2.1)	18.5 (2.2)	0.190	0.002	0.003
FVC (L)	3.6 (0.9)	3.9 (0.9)	0.003	3.2 (0.8)	3.3 (0.7)	0.024	0.003	0.002
FEV1 (L)	2.7 (0.7)	3.2 (0.7)	0.001	2.5 (0.5)	2.4 (0.5)	0.211	0.001	0.001
IT (%)	77.0 (5.0)	80.3 (5.3)	0.001	77.1 (9.3)	74.3 (7.8)	0.131	0.002	0.002

BMI = Body Mass Index; HbA1c = glycated hemoglobin; glycemia; Col TOT = total cholesterol; HDL = high density lipoprotein; LDL = low density lipoprotein; Tg = triglycerides: VO_2_ peak = maximum oxygen uptake; FVC = forced vital capacity; FEV1 = forced expiratory volume in one second; IT = Tiffenau index (FEV1/FVC).

## References

[1] Steel Z., Marnane C., Iranpour C., Chey T., Jackson J.W., Patel V., Silove D. (2014). The global prevalence of common mental disorders: A systematic review and meta-analysis 1980–2013. Int. J. Epidemiol..

[2] Evans S., Banerjee S., Leese M., Huxley P. (2007). The impact of mental illness on quality of life: A comparison of severe mental illness, common mental disorder and healthy population samples. Qual. Life Res..

[3] Bloom D.E., Cafiero E.T., Jané-Llopis E., Abrahams-Gessel S., Bloom L.R., Fathima S., Feigl A.B., Gaziano T., Hamandi A., Mowafi M. (2010). The Global Economic Burden of Noncommunicable Diseases.

[4] James S.L., Abate D., Abate K.H., Abay S.M., Abbafati C., Abbasi N., Abbastabar H., Abd-Allah F., Abdela J., Abdelalim A. (2018). Disease and Injury Incidence and Prevalence Collaborators. Global, regional, and national incidence, prevalence, and years lived with disability for 354 diseases and injuries for 195 countries and territories, 1990–2017: A systematic analysis for the Global Burden of Disease Study 2017. Lancet.

[5] Ohrnberger J., Fichera E., Sutton M. (2017). The relationship between physical and mental health: A mediation analysis. Soc. Sci. Med..

[6] Stein D.J., Benjet C., Gureye O., Lund C., Scott K.M., Poznyak V., Van Ommeren M. (2019). Integrating mental health with other non-communicable diseases. BMJ.

[7] Smidt N., de Vet H.C.W., Bouter L.M., Dekker J., Arendzen J.H., de Bie R.A., Bierma-Zeinstra S.M., Helders P.J., Keus S.H., Kwakkel G. (2005). Effectiveness of exercise therapy: A best-evidence summary of systematic reviews. Aust. J. Physiother..

[8] Peiris C.L., Taylor N.F., Shields N. (2011). Extra physical therapy reduces patient length of stay and improves functional outcomes and quality of life in people with acute or subacute conditions: A systematic review. Arch. Phys. Med. Rehabil..

[9] Palermi S., Iacono O., Sirico F., Modestino M., Ruosi C., Spera R., De Luca M. (2021). The complex relationship between physical activity and diabetes: An overview. J. Basic. Clin. Physiol. Pharmacol..

[10] Palermi S., Sacco A.M., Belviso I., Marino N., Gambardella F., Loiacono C., Sirico F. (2020). Effectiveness of Tai Chi on Balance Improvement in Type 2 Diabetes Patients: A Systematic Review and Meta-Analysis. J. Aging Phys. Act..

[11] Palermi S., Bragazzi N., Cular D., Ardigò L., Padulo J. (2021). How chest press-based exercises can alleviate the burden of cardiovascular diseases. Hum. Mov..

[12] Rosenbaum S., Tiedemann A., Sherrington C., Curtis J., Ward P.B. (2014). Physical activity interventions for people with mental illness: A systematic review and meta-analysis. J. Clin. Psychiatry.

[13] Malhi G.S., Bassett D., Boyce P.M., Bryant R., Fitzgerald P.B., Fritz K., Hopwood M., Lyndon B., Mulder R., Murray G. (2015). Royal Australian and New Zealand College of Psychiatrists clinical practice guidelines for mood disorders. Aust. N. Z. J. Psychiatry.

[14] Stephen R., Hongisto K., Solomon A., Lönnroos E. (2017). Physical Activity and Alzheimer’s Disease: A Systematic Review. J. Gerontol. A Biol. Sci. Med. Sci..

[15] Zschucke E., Gaudlitz K., Ströhle A. (2013). Exercise and physical activity in mental disorders: Clinical and experimental evidence. J. Prev. Med. Public Health.

[16] Lamberti V., Nardini S., Romano P., Menegon T., Lamberti V.S. (2015). Una nuova frontiera nella sport-terapia: AMPA system (attività motoria personalizzata e adattata). Med. Dello Sport.

[17] Radtke T., Crook S., Kaltsakas G., Louvaris Z., Berton D., Urquhart D.S., Kampouras A., Rabinovich R.A., Verges S., Kontopidis D. (2019). ERS statement on standardisation of cardiopulmonary exercise testing in chronic lung diseases. Eur. Respir. Rev..

[18] Ware J.E., Kosinski M., Keller S.D. (1996). A 12-Item Short-Form Health Survey: Construction of Scales and Preliminary Tests of Reliability and Validity. Med. Care.

[19] Liguori G., Kluwer W. (2021). ACSMs Guidelines for Exercise Testing and Prescription.

[20] Wenger N.K., Froelicher E.S., Smith L.K., Philip A., Ades P.A., Berra K., Blumenthal A.J., Certo C.M., Dattilo A.M., Davis D. (1995). Cardiac Rehabilitation as Secondary Prevention—Clinical Practice Guidelines n°17.

[21] Thompson P.D. (2005). Exercise prescription and proscription for patients with coronary artery disease. Circulation.

[22] Durstine J.L., Moore G.E., Painter P.L., Roberts S.O. (2009). ACSM’s Exercise Management for Persons with Chronic Diseases and Disabilities.

[23] Probst M. (2017). Psychomotor Therapy for Patients with Severe Mental Health Disorders, in Occupational Therapy—Occupation Focused Holistic Practice in Rehabilitation.

[24] Chekroud S.R., Gueorguieva R., Zheutlin A.B., Paulus M., Krumholz H.M., Krystal J.H., Chekroud A.M. (2018). Association between physical exercise and mental health in 1·2 million individuals in the USA between 2011 and 2015: A cross-sectional study. Lancet Psychiatry.

[25] Kerling A., Tegtbur U., Gützlaff E., Kück M., Borchert L., Ates Z., von Bohlen A., Frieling H., Hüper K., Hartung D. (2015). Effects of adjunctive exercise on physiological and psychological parameters in depression: A randomized pilot trial. J. Affect. Disord..

[26] Knapen J., Vancampfort D., Moriën Y., Marchal Y. (2015). Exercise therapy improves both mental and physical health in patients with major depression. Disabil. Rehabil..

[27] Warburton D.E.R., Bredin S.S.D. (2017). Health benefits of physical activity: A systematic review of current systematic reviews. Curr. Opin. Cardiol..

[28] Lakicevic N., Gentile A., Mehrabi S., Cassar S., Parker K., Roklicer R., Bianco A., Drid P. (2020). Make Fitness Fun: Could Novelty Be the Key Determinant for Physical Activity Adherence?. Front. Psychol..

[29] Schuch F.B., Vancampfort D., Richards J., Rosenbaum S., Ward P.B., Stubbs B. (2016). Exercise as a treatment for depression: A meta-analysis adjusting for publication bias. J. Psychiatr. Res..

[30] Danielsson L., Kihlbom B., Rosberg S. (2016). ‘Crawling Out of the Cocoon’: Patients’ Experiences of a Physical Therapy Exercise Intervention in the Treatment of Major Depression. Phys. Ther..

[31] Swift D.L., Johannsen N.M., Lavie C.J., Earnest C.P., Church T.S. (2014). The role of exercise and physical activity in weight loss and maintenance. Prog. Cardiovasc. Dis..

[32] Slentz C.A., Duscha B.D., Johnson J.L., Ketchum K., Aiken L.B., Samsa G.P., Houmard J.A., Bales C.W., Kraus W.E. (2004). Effects of the Amount of Exercise on Body Weight, Body Composition, and Measures of Central Obesity: STRRIDE—A Randomized Controlled Study. Arch. Intern. Med..

[33] Ma C., Avenell A., Bolland M., Hudson J., Stewart F., Robertson C., Sharma P., Fraser C., MacLennan G. (2017). Effects of weight loss interventions for adults who are obese on mortality, cardiovascular disease, and cancer: Systematic review and meta-analysis. BMJ.

[34] Knopp R.H. (1999). Drug treatment of lipid disorders. N. Engl. J. Med..

[35] Boulé N.G., Kenny G.P., Haddad E., Wells G.A., Sigal R.J. (2003). Meta-analysis of the effect of structured exercise training on cardiorespiratory fitness in Type 2 diabetes mellitus. Diabetologia.

[36] Cauza E., Hanusch-Enserer U., Strasser B., Kostner K., Dunky A., Haber P. (2005). Strength and endurance training lead to different post exercise glucose profiles in diabetic participants using a continuous subcutaneous glucose monitoring system. Eur. J. Clin. Investig..

[37] Colberg S.R., Sigal R.J., Yardley J.E., Riddell M.C., Dunstan D.W., Dempsey P.C., Horton E.S., Castorino K., Tate D.F. (2016). Physical Activity/Exercise and Diabetes: A Position Statement of the American Diabetes Association. Diabetes Care.

[38] Ross R., Janssen I., Dawson J., Kungl A.M., Kuk J.L., Wong S.L., Nguyen-Duy T.B., Lee S., Kilpatrick K., Hudson R. (2004). Exercise-induced reduction in obesity and insulin resistance in women: A randomized controlled trial. Obes. Res..

[39] Pescatello L.S., Franklin B.A., Fagard R., Farquhar W.B., Kelley G.A., Ray C.A., American College of Sports Medicine (2004). American College of Sports Medicine position stand. Exercise and hypertension. Med. Sci. Sports Exerc..

[40] Biffi A., Gallo G., Fernando F., Sirico F., Signorello M.G., De Martino L., Manole G.E., Palermi S., Volpe M. (2022). Relationship between Cardiorespiratory Fitness, Baseline Blood Pressure and Hypertensive Response to Exercise in the Ferrari Corporate Population. High Blood Press Cardiovasc. Prev..

[41] Cooper C.B. (1995). Determining the role of exercise in patients with chronic pulmonary disease. Med. Sci. Sports Exerc..

[42] Salman G.F., Mosier M.C., Beasley B.W., Calkins D.R. (2003). Rehabilitation for patients with chronic obstructive pulmonary disease: Meta-analysis of randomized controlled trials. J. Gen. Intern. Med..

[43] Scherer T.A., Spengler C.M., Owassapian D., Imhof E., Boutellier U. (2000). Respiratory muscle endurance training in chronic obstructive pulmonary disease: Impact on exercise capacity, dyspnea, and quality of life. Am. J. Respir. Crit. Care Med..

[44] Palermi S., Massa B., Vecchiato M., Mazza F., De Blasiis P., Romano A.M., Di Salvatore M.G., Della Valle E., Tarantino D., Ruosi C. (2021). Indirect Structural Muscle Injuries of Lower Limb: Rehabilitation and Therapeutic Exercise. J. Funct. Morphol. Kinesiol..

[45] Belviso I., Palermi S., Sacco A.M., Romano V., Corrado B., Zappia M., Sirico F. (2020). Brachial Plexus Injuries in Sport Medicine: Clinical Evaluation, Diagnostic Approaches, Treatment Options, and Rehabilitative Interventions. J. Funct. Morphol. Kinesiol..

